# Over-expression of mitochondrial antiviral signaling protein inhibits coxsackievirus B3 infection by enhancing type-I interferons production

**DOI:** 10.1186/1743-422X-9-312

**Published:** 2012-12-19

**Authors:** Qing-Meng Zhang, Wu-Qi Song, Yu-Jun Li, Jun Qian, Ai-Xia Zhai, Jing Wu, Ai-Mei Li, Jun-Ming He, Jin-Yun Zhao, Xin Yu, Lan-Lan Wei, Feng-Min Zhang

**Affiliations:** 1 The Key Laboratory of Immunity and Infection, Pathogenic Biology, Heilongjiang province, Department of Microbiology, Harbin Medical University, 150081, Harbin, People’s Republic of China; 2 Bio-pharmaceutical Key Laboratory, Harbin Medical University, Ministry of Education of China, Harbin, 150081, People’s Republic of China

**Keywords:** Mitochondrial antiviral signaling protein, Coxsackievirus B3, Interferon, Antiviral response

## Abstract

**Background:**

Recent studies have revealed that Mitochondrial Antiviral Signaling (MAVS) protein plays an essential role in the inhibition of viral infection through type I interferon (IFN) pathway. It has been shown that 3C (pro) cysteine protease of coxsackievirus B3 (CVB3) cleaves MAVS to inhibit type I IFNs induction. Other workers also found that MAVS knock-out mice suffered CVB3 susceptibility and severe histopathological change. Accordingly,our experiments were designed to explore the protection of over-expressing MAVS against CVB3 infection and the possible mechanism.

**Results:**

In this study, HeLa cells (transfected with MAVS constructs pre- or post- exposure to CVB3) were used to analyze the function of exogenous MAVS on CVB3 infection. The results revealed that though CVB3 infection induced production of type I IFNs, viral replication and cell death were not effectively inhibited. Similarly, exogenous MAVS increased type I IFNs moderately. Morever, we observed robust production of type I IFNs in CVB3 post-infected HeLa cells thereby successfully inhibiting CVB3 infection, as well formation of cytopathic effect (CPE) and cell death. Finally, introduction of exogenous MAVS into CVB3 pre-infected cells also restricted viral infection efficiently by greatly up-regulating IFNs.

**Conclusions:**

In summary, exogenous MAVS effectively prevents and controls CVB3 infection by modulating and promoting the production of type I IFNs. The IFNs level in MAVS over-expressing cells is still tightly regulated by CVB3 infection. Thus, the factors that up-regulate MAVS might be an alternative prescription in CVB3-related syndromes by enhancing IFNs production.

## Background

MAVS is an important type I IFNs-related protein and a common adaptor molecule of RIG-I/MDA5 [1-4]. This protein is localized on the mitochondria outer membrane through its C-terminal transmembrane domain. Viral infection triggers the activation of RIG-I/MDA5 which recruits MAVS by the tandem caspases activation and recruitment domains (CARDs). Then MAVS links RIG-I/MDA5 to IKK and TBK1/IKKε and subsequently activates IRF3 and NFκB which are both key factors in the induction pathway of IFNs in response to viral infection [5-7].

IFN-β induction by MDA5/RIG-I–MAVS pathway plays an essential role in infection of Rotavirus, Encephalomyocarditis virus, Measles virus and Newcastle virus [8-12]. Over-expression of MAVS activates NF-κB and IRF3 and induces type I IFNs, resisting infection of Vesicular Stomatitis Virus (VSV) and Sendai Virus (SeV) [3,4,10,13]. On the contrary, knockdown of endogenous MAVS blocks the induction of type I IFNs initiated by polyriboinosinic polyribocytidylic acid (poly I:C) or virus, leading to enhanced susceptibility to viral infection [1,3]. Blocking MAVS expression resulted in obvious cell damage when exposed to SeV, VSV or Newcastle virus [14,15]. Other data reveal that over-expression of MAVS in Teleost fish cells led to induction of IFNs and IFN-stimulated genes (ISGs). MAVS over-expression not only almost fully protected the fish cells against RNA virus infection but also strongly inhibited both DNA and RNA virus replication [16].

CVB3, a member of *picornavirus* family, is a common human pathogen and is associated with diverse syndromes such as viral myocarditis, diabetes, meningitis and febrile illnesses [17-19]. CVB3 infects host cells through coxsackievirus and adenovirus receptor (CAR) and replicates in cytoplasm causing tissue damage. The absence of MAVS leads to deficient type I IFNs production and early mortality in mice infected with CVB, and the subsequent histopathological examination have shown pancreatic and hepatic necrosis [20], which highlights the critical role of MAVS in cell protection against CVB3. A recent study has demonstrated that the 3C (pro) cysteine protease of CVB3 cleaves MAVS and Toll/IL-1 receptor domain-containing adaptor inducing IFN-β (TRIF) to inhibit the induction of type I IFNs [21], which may influence immune clearance, permit viral persistence and cause immunopathologic damage [13,22]. But whether over-expression of MAVS in host cells inhibits CVB3 infection still remains unreported.

In this study, pre- or post- infection with CVB3, exogenous MAVS was introduced into HeLa cells and the anti-CVB3 activity and mechanism of MAVS were investigated. Our results showed that type I IFNs produced due to CVB3 infection was insufficient to inhibit viral replication or cell damage. Although exogenous MAVS resulted in moderate IFNs induction, CVB3 infection initiated robust production of endogenous type I IFNs in HeLa cells over-expressing MAVS, with obvious concurrent decline of CVB3 replication. Introduction of MAVS into CVB3 infected HeLa cells could also help restrict the virus replication. Our results demonstrate that the exogenous MAVS enhanced innate immune plays a pivotal role in anti-CVB3 process.

## Results

### Influence of exogenous MAVS on cell growth and IFN production

**Figure 1:**
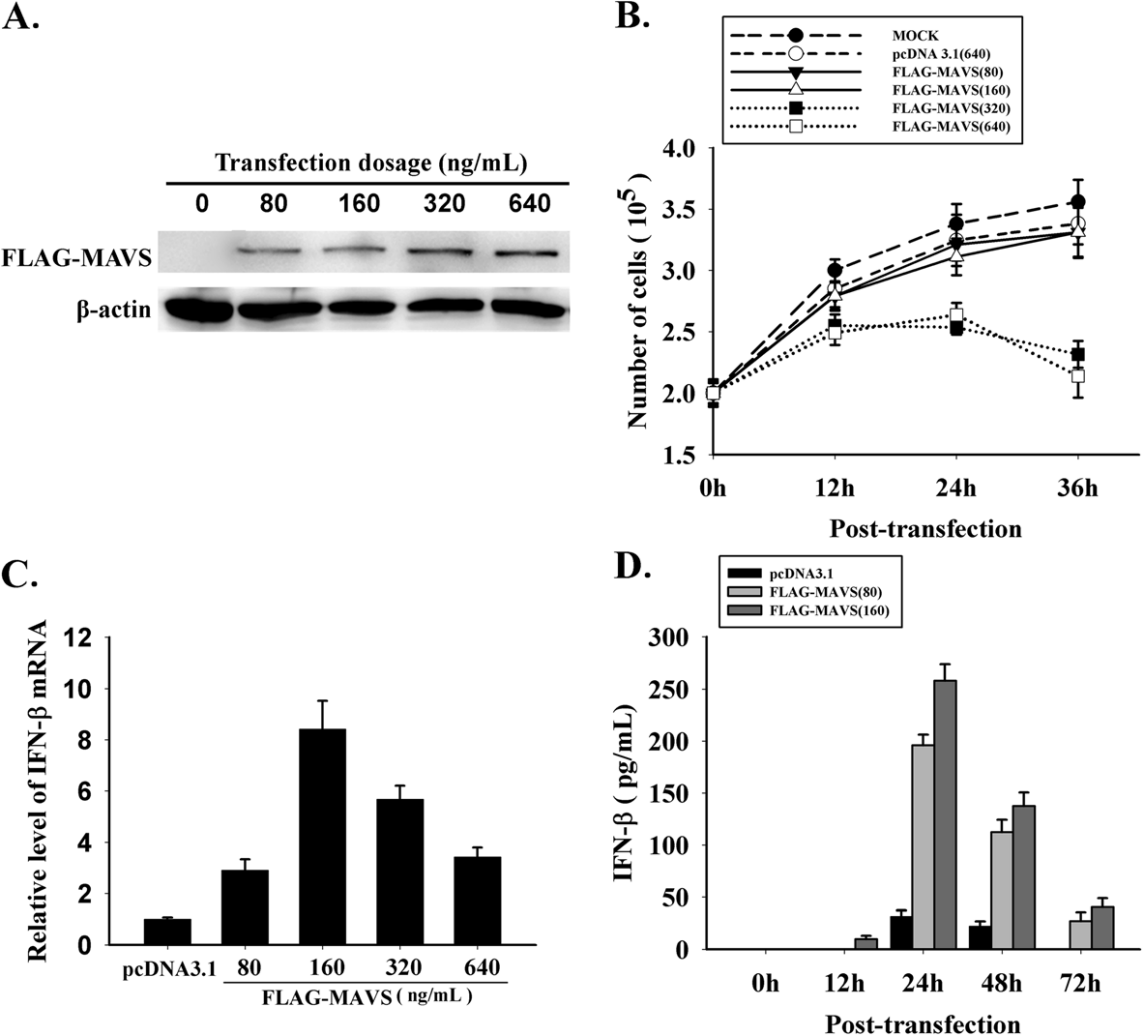
**Time and dose effect of MAVS on cell growth and IFN-β production.** HeLa cells were transfected with pcDNA 3.1 and pcDNA3.1-FLAG-MAVS. (**A**) At 24 h post-transfection, fusion protein of FLAG-MAVS was detected by western blotting. (**B**) Cell proliferation was evaluated through trypan blue dye exclusion. (**C**) At 24 h post-transfection, RT-qPCR was performed to evaluate the IFN-β transcriptional level at different dosages of MAVS construct transfection. (**D**) The concentration of IFN-β in culture supernatant was determined with ELISA at different time points post-transfection. * *p* < 0.05,** *p* < 0.01.

HeLa cells were transfected with pcDNA3.1 or pcDNA3.1-FLAG-MAVS (80 ng/mL, 160 ng/mL, 320 ng/mL, 640 ng/mL), and the fusion protein FLAG-MAVS was detected by Western Blotting at 24 h post-transfection. Increased transfection efficiency was observed in a pcDNA3.1-FLAG-MAVS dose dependent manner in the HeLa cells (Figure 1A). The number of viable cells was counted by trypan blue dye exclusion using a hemocytometer to determine the influence of plasmid dose upon cell growth. The results suggested that the cells, in MAVS group at the dose of 80 ng/mL or 160 ng/mL, shared the similar growth level with those in empty vector group (Figure 1B). To detect the induction of IFN-β by different doses of exogenous MAVS, cells were harvested at 24 h post-transfection, total RNA was extracted and then RT-qPCR was performed (Figure 1C). The results showed that dose of 160 ng/mL induced more IFN-β transcription than other groups. Therefore we used the two doses of MAVS plasmid (80 ng/mL & 160 ng/mL) to transfect HeLa cells, and harvested the culture supernatant at 0 h, 12 h, 24 h, 48 h and 72 h post-transfection. The concentration of IFN-β was determined by ELISA (Figure 1D). Our data indicated that production of IFN-β peaked at 24 h and began to decline during 24 h to 48 h. Those results showed that with suitable introduction dose, exogenous MAVS is low-toxic or even non-toxic, and the production of IFN-β initiated by over-expressing MAVS is time- and dose-dependent.

### Inhibition of CVB3 in MAVS over-expressing cells

**Figure 2:**
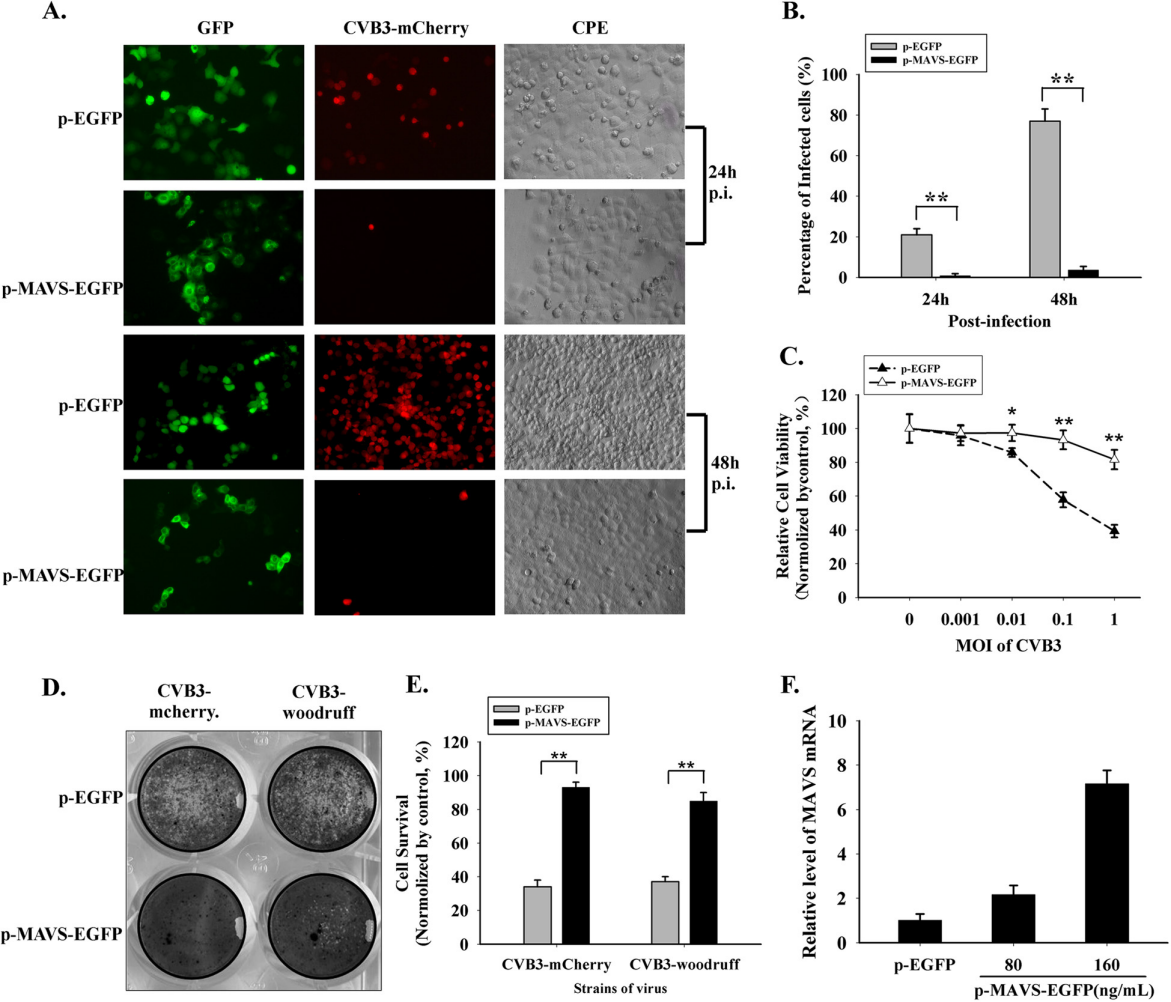
**Restriction of CVB3 caused CPE by exogenous MAVS in HeLa cells.** At 24 h post-transfected with p-EGFP or p-MAVS-EGFP, HeLa cells were challenged by CVB3-mCherry. After 24 h and 48 h of CVB3-mCherry infection, (**A**) the expression of MAVS-GFP (green), CVB3-mCherry infection (red) and the CPE were observed. (**B**) The percentage of infected cells was calculated. (**C**) CVB3-mCherry was used to inoculate HeLa cells (MOI = 0, 0.001, 0.01, 0.1, 1) after transfection with p-EGFP or p-MAVS-EGFP. MTT test was used to determine the cell viability. (**D** &**E**) Cells over-expressing exogenous MAVS were exposed to CVB3-woodruff or CVB3-mCherry respectively, and the survival cells were stained by crystal violet. The percentage of survival cells was evaluated by observation. (**F**) HeLa cells were transfected with p-EGFP or p-MAVS-EGFP for 24 h and harvested for RNA extraction, and RT-qPCR was performed to verify the expression level of exogenous MAVS. * *p* < 0.05,** *p* < 0.01.

HeLa cells were challenged with CVB3-mCherry (MOI = 0.01) at 24 h post-transfection with p-EGFP or p-MAVS-EGFP (160 ng/mL). At 24 h and 48 h after CVB3-mCherry inoculation, the expression of MAVS-GFP fusion protein and mCherry protein were observed through fluorescence microscope. Simultaneously the CPE caused by CVB3-mCherry was observed through optical microscope. The fusion protein MAVS-GFP presented disperse and ununiform distribution in cytoplasm, while the GFP expressed by p-EGFP-N1 vector was localized evenly in nuclear and cytosolic space (Figure 2A). In p-EGFP group,at 24 h post-infection, over 20% cells expressed visible red fluorescence and part of cells suffered CPE, and at 48 h post-inoculation, 70% ~ 80% cells were red and the CPE became more severe (Figure 2B). On the contrary, in the MAVS group, no red fluorescence was visible at 24 h post-infection and only individual red spots could be observed at 48 h but without obvious CPE (Figure 2A and B). In another experiment, we transfected HeLa cells with p-EGFP and p-MAVS-EGFP (160 ng/mL), and challenged them with CVB3-mCherry at MOI of 0, 0.001, 0.01, 0.1 and 1. At 48 h post-infection, cell viability was determined by MTT test. The cells mortality in empty vector group was significantly higher than MAVS group (Figure 2C). After this, CVB3-Woodruff and CVB3-mCherry, the two CVB3 strains, were used to inoculate (MOI = 0.01) HeLa cells transfected with p-EGFP and p-MAVS-EGFP (160 ng/mL). Upon culturing for 48 h, the cells were stained with crystal violet. The results showed that the CVB3-Woodruff and CVB3-mCherry caused similar CPE in HeLa cells, and MAVS construct plasmid successfully protected cells against cell death caused by both the virus strains (Figure 2D and E). Those findings indicated that exogenous MAVS pre-treatment obviously inhibits the cell damage caused by CVB3 infection. To verify the MAVS protein expressed by p-MAVS-EGFP, HeLa cells were harvested at 24 h post-transfection, and RT-qPCR was performed (Figure 2F). Comparing with the constitutive MAVS expression in HeLa cells, introduction of the new construct plasmid increased the MAVS level obviously.

### Inhibition of CVB3 by over-expressing MAVS coincides with type I IFNs increase

**Figure 3:**
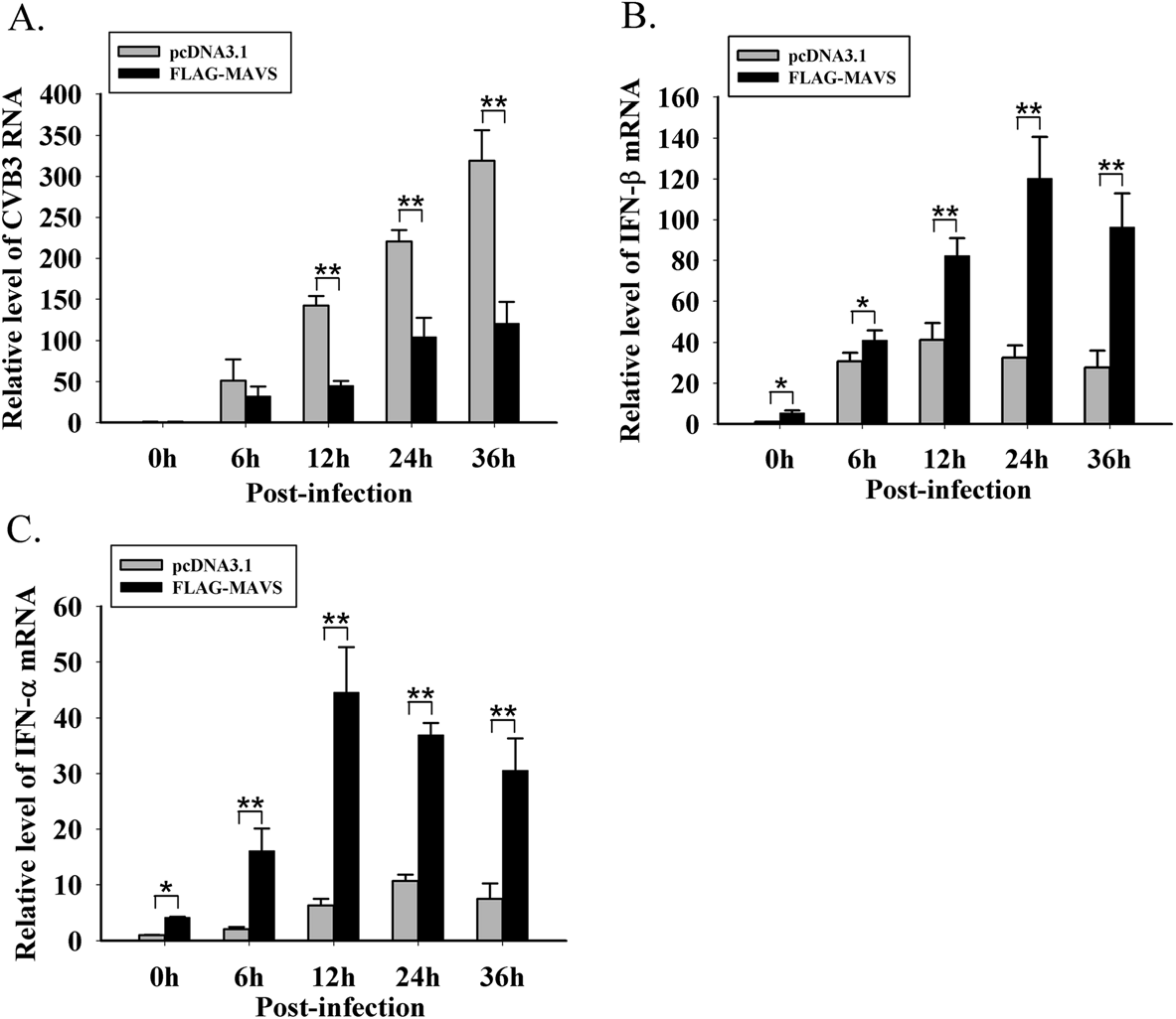
**Inhibition of CVB3 by over-expressing MAVS, coinciding with IFNs increase.** After transfection with pcDNA3.1 and pcDNA3.1-FLAG-MAVS, HeLa cells were infected with CVB3-woodruff. At 0 h, 6 h, 12 h, 24 h and 36 h post-infection, cells were harvested, and RT-qPCR was performed to determine the change of (**A**) CVB3 replication, (**B**) IFN-β mRNA and (**C**) IFN-α mRNA. * *p* < 0.05,** *p* < 0.01.

HeLa cells were challenged by CVB3-woodruff (MOI = 0.01) at 24 h post-transfection with pcDNA3.1 and pcDNA3.1-FLAG-MAVS (160 ng/mL). Cells were harvested at 0 h, 6 h, 12 h, 24 h and 36 h after infection and RT-qPCR was performed to evaluate the replication level of CVB3 and the transcriptional level of IFN-α/β. In the pcDNA3.1 group, during 6 h to 36 h post-infection, the CVB3 replication increased over 6-fold (Figure 3A), whereas the IFN-α/β were only up-regulated mildly (Figure 3B and C). From 12 h to 36 h, the CVB3 level in FLAG-MAVS group was lower comparing to the pcDNA3.1 group with statistical significance (Figure 3A). This pattern coincided with which the transcriptional levels of IFN-α/β in MAVS group (Figure 3B and C). In FLAG-MAVS group, the IFN-α/β expression showed a significant increase after CVB3 infection and the expression level peaked during 12 h to 24 h post-infection (Figure 3B and C). The findings suggest that the IFNs induced by CVB3 infection are insufficient to restrict viral replication, but exogenous MAVS could promote the endogenous production of IFNs in the infected cells and successfully restrict CVB3 replication.

### Over-expressing MAVS efficiently reduces the existing CVB3 infection by up-regulation of type I IFNs

**Figure 4:**
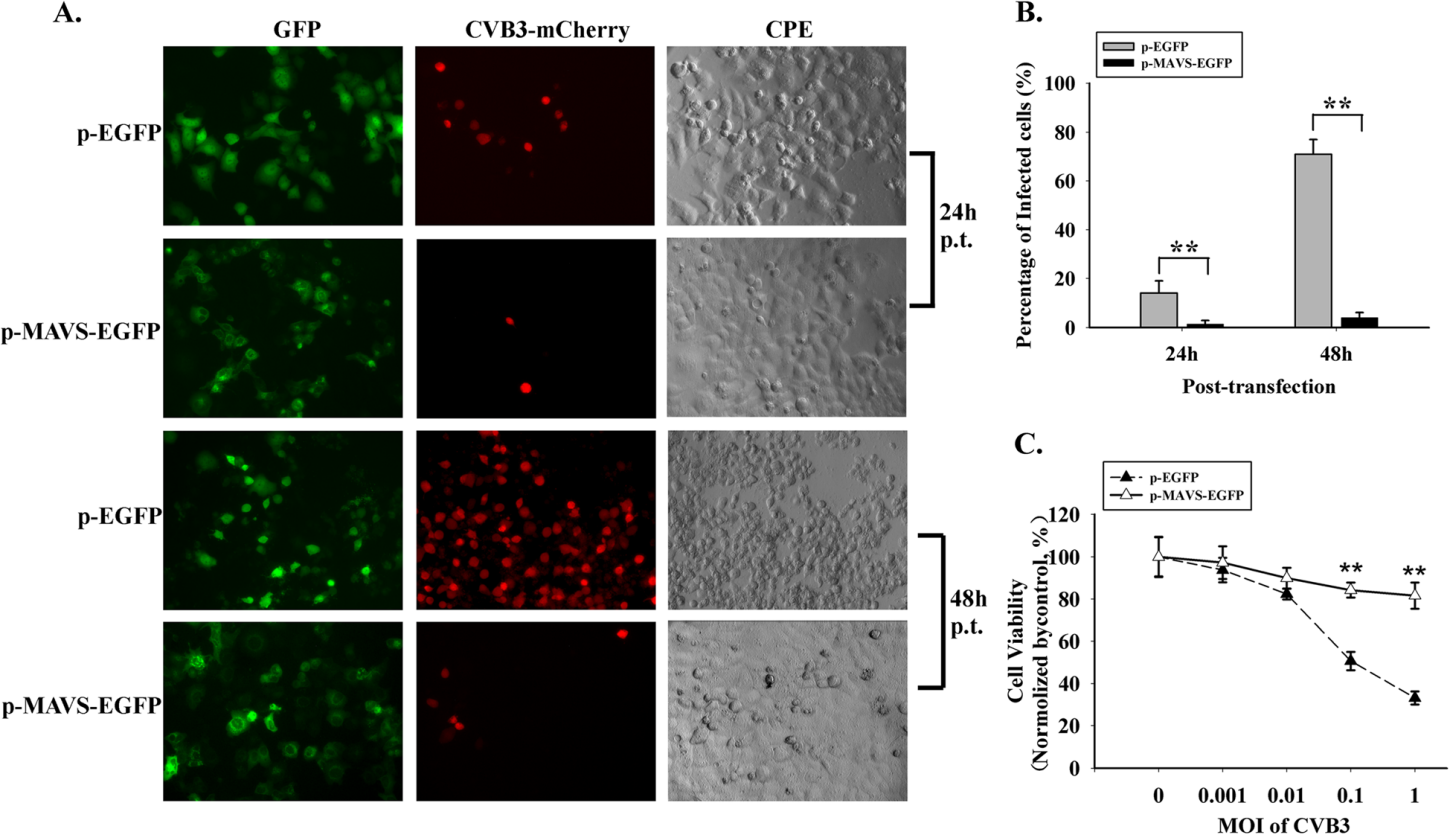
**Inhibition of CVB3 by exogenous MAVS in pre-infected HeLa cells.** HeLa cells were challenged by CVB3-mCherry (absorption for 1 h) and then transfected with p-EGFP and p-MAVS-EGFP. (**A**) MAVS-GFP expression (green), CVB3 infection (red) and CPE were observed at 24 h and 48 h. (**B**) The percentage of infected cells with red fluorescence was calculated. (**C**) MTT was performed to determine the cell viability of HeLa cells inoculated with CVB3 at MOI of 0, 0.001, 0.01, 0.1, 1 and then transfected with p-EGFP and p-MAVS-EGFP plasmids (48 h post-transfection). * *p* < 0.05,** *p* < 0.01.

After exposure to CVB3-mCherry for 1 h (MOI = 0.01), HeLa cells were transfected with p-EGFP or p-MAVS-EGFP (160 ng/mL). At 24 h and 48 h post-transfection, the expression of MAVS-GFP fusion protein and infective efficiency of CVB3-mCherry were observed through fluorescence microscope. The CPE caused by CVB3 was observed through optical microscope (Figure 4A). The results showed that in p-EGFP group 15% at 24 h and 70% at 48 h of cells expressed visible red fluorescence protein and obvious CPE was observed. In contrast, in MAVS-GFP group, only 2% at 24 h and 4% at 48 h of cells expressed red fluorescence protein (Figure 4B). At 48 h after CVB3 inoculation with MOI grades (0, 0.001, 0.01, 0.1 and 1), viral absorption for 1 h and transfection with p-EGFP and p-MAVS-EGFP (160 ng/mL), cell viability was evaluated with MTT test. As seen in Figure 2E, we found that the cell death in MAVS group is milder than in empty vector group (Figure 4C).

**Figure 5:**
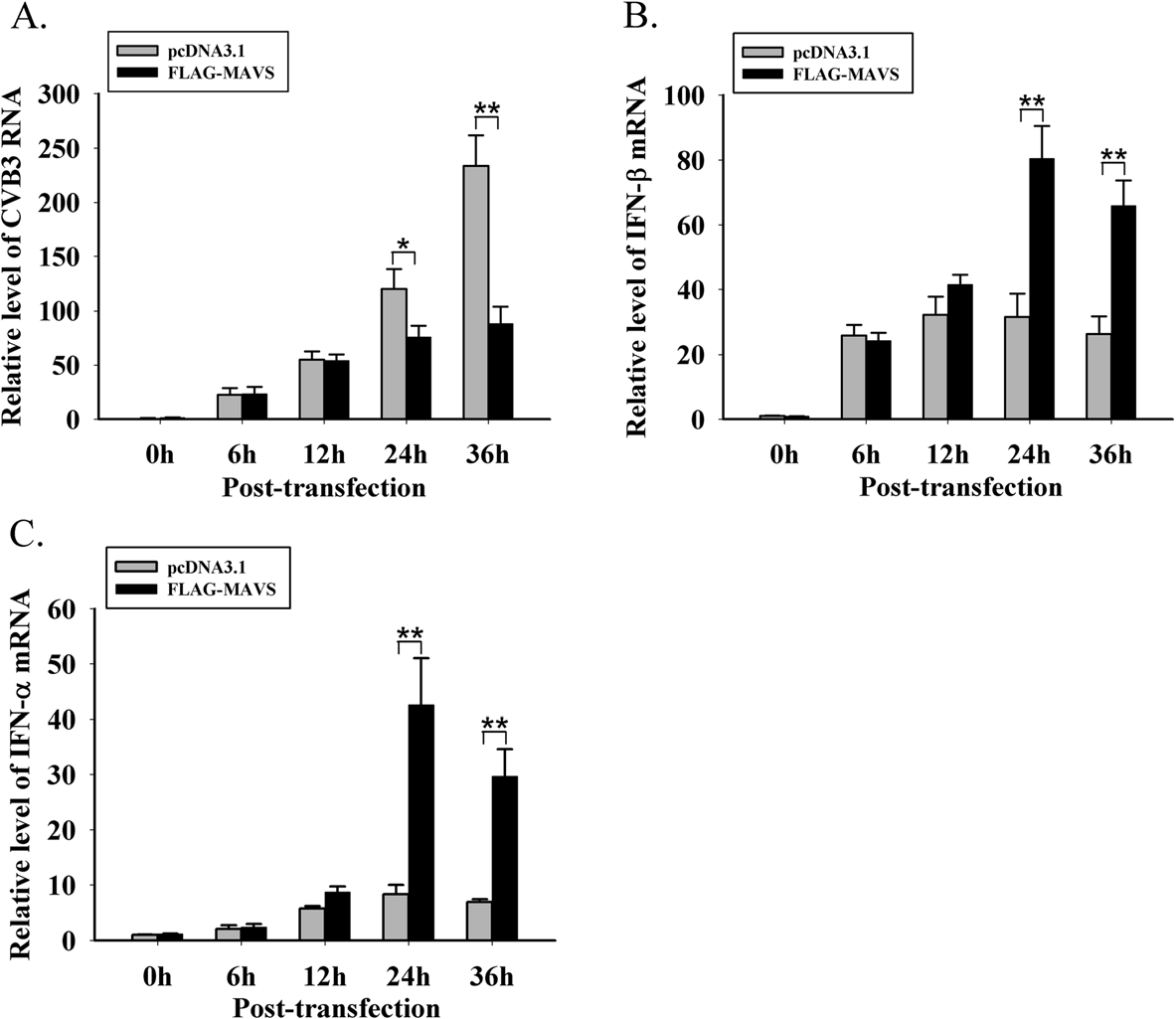
**Inhibition of CVB3 and up-regulation of IFNs by MAVS introduction after CVB3 infection.** After exposure to CVB3 for 1 h, pcDNA3.1 and pcDNA3.1-FLAG-MAVS were introduced into HeLa cells. In 0 h ~ 36 h, cells were harvested and total RNA was extracted. (**A**) CVB3 RNA, (**B**) IFN-β mRNA and (**C**) IFN-α mRNA were detected through RT-qPCR. * *p* < 0.05,** *p* < 0.01.

After exposure to CVB3-woodruff for 1 h (MOI = 0.01),HeLa cells were transfected with pcDNA3.1 and pcDNA3.1-FLAG-MAVS (160 ng/mL) and harvested at 0 h, 6 h, 12 h, 24 h and 36 h post-transfection. RT-qPCR was performed to evaluate the replication level of CVB3 and the expression levels of IFN-α/β. The data showed that from 0 h to12 h, there was no significant difference of CVB3 replication between MAVS group and empty vector group. During 24 h to 36 h, in the samples harvested at the same time points, CVB3 replication in empty vector group was significantly higher than MAVS group (Figure 5A). The IFN-α/β mRNAs in MAVS group began to increase at 12 h and peaked at 24 h (Figure 5B and C). Along with increase of type I IFNs expression, the CVB3 replication was restricted significantly in MAVS group. These results indicated that even in pre-infected cells, exogenous MAVS still inhibits CVB3 infection successfully, and the up-regulation of type I IFNs is involved in the viral inhibition.

## Discussion

Type I IFNs play an important part in resistance of viral infection. The double strand RNA (dsRNA) and single strand RNA (ssRNA), as the by-products of viral replication, were recognized by Toll like receptors (TLRs) on cell plasma membrane or RIG-I/MDA5 in cytoplasm [23,24]. The induction signal is transferred to TRIF or MAVS and activates IRF3 and NFκB, which play critical roles in production of type I IFNs [5,6]. In recent years, many studies have focused on the pivotal role of MAVS in antiviral innate immune.

After introducing MAVS plasmid into HeLa cells and then inoculating the cells with CVB3, we found that the replication of CVB3 was inhibited and the virus-related cell death also decreased. We further introduced MAVS into HeLa cells, just after exposure to CVB3. The subsequent results revealed that either way (before or after exposure to CVB3), the cells were efficiently protected against viral infection by robust up-regulation of type I IFNs expression. When CVB3 was used to challenge HeLa cells without exogenous MAVS construct plasmid, we found that although IFN-α/β mRNAs were up-regulated, the highly raised CVB3 replication and CPE indicated that type I IFNs induced by CVB3 infection are insufficient to restrict CVB3 replication.

Even in MAVS over-expressing cells, the IFNs production is regulated by CVB3 infection to a great extent. Our results demonstrated that exogenous MAVS could induce type I IFNs, which is consistent with previous reports. We found that when the cells expressing exogenous MAVS protein were challenged with CVB3, the induction of type I IFNs increased more robustly than the unchallenged group. To the best of our knowledge, excessive IFN expression may cause negative effects to the host, such as autoimmune disorders and activation of apoptosis. Therefore, to keep the host from such disorders, accurate control of IFN induction is necessary, moderate expression to suit physiological condition, but still retain sufficient levels for anti-pathogens defense. Recent research have revealed that in HEK293T cell line stably expressing FLAG-MAVS, the FLAG-MAVS fusion protein purified from the virus-infected cells formed aggregates on mitochondrial membrane and was capable of activating IRF3 much more than that from the mock-infected cells [25]. These results suggest that the activation of MAVS associated IFN pathway is tightly regulated by viral infection, which might beneficially contribute to accurate control of immune response.

The process of viral infection includes not only the host defense against virus but also the negative influence of virus to host defense. Type I IFNs play an important role in host defense, such as inhibiting viral replication directly and mediating the cross-link between innate and adaptive immune. However, when certain virus infects host cells, type I IFNs induction is inhibited by viral components, such as the NS1 of influenza virus, the NS3/4A of Hepatitis C virus and the P protein of Borna Disease Virus [26-28]. Eventually the competition between host defense and viral retroaction determines the status of viral infection. Many findings have pointed to the mutual influence and restriction between viral replication and host immune response. MAVS or MDA5 knockout mice with impaired type I IFNs pathway have been showed to suffer more serious histological necrosis and higher mortality than the wild type mice after exposure to CVB3 [20], which demonstrates the critical role of MAVS in anti-CVB3 infection. As seen in many other viruses, CVB3 components also interact with innate immune pathway. In CVB3 infected HeLa cell line, MAVS protein was cleaved by the 3C (pro) cysteine protease of CVB3 [21]. Thus, deficiency or decrease of MAVS may result in non-clearance of CVB3 and the subsequent tissue necrosis, viral persistence or immunopathologic damage, with a latent possibility of CVB3 related symptoms such as viral myocarditis [13,22]. In our study, even though CVB3 infection induced IFNs, the amount of those antiviral cytokines could not restrict CVB3 infection in return. But, CVB3 replication and virus caused cell death were both restricted in MAVS-transfected group. Therefore we infer that over-expression of MAVS in HeLa cells recovers the impaired MAVS-IFNs pathway to restrict CVB3 infection. Besides, CVB3 infection was also influenced by other factors such as expression of CAR, the essential receptor for CVB3 on membrane of permissive cells. In another experiment, our results demonstrated that exogenous MAVS does not change the expression of CAR (Data not shown). Thus it could be concluded that the inhibition of CVB3 by MAVS is independent of CAR regulation.

Control of CVB3 infection is one of the efficient clinical therapeutic methods to relieve virus associated syndromes like viral myocarditis caused by CVB3. Type I IFNs, especially IFN-α 2a and IFN-α 2b, are widely used in treatment of CVB3 infection [29]. It was reported that IFN-β inhibits CVB3 infection more efficiently than IFN-α [7,30]. Poly I:C, an important synthetic inducers of IFNs, has also been applied in adjuvant treatment of viral infection and tumor [31]. In laboratory and clinical research, poly I:C inhibits CVB3 infection efficiently through induction of type I IFNs [32,33]. But, intriguingly, MAVS, which is pivotal in poly I:C anti-virus activities, is cleaved by 3C (pro) cysteine protease of CVB3. Taken those together, we infer that poly I:C may initiate the innate immune response as well up-regulate the expression of MAVS to overcome the protein reduction by CVB3. In a earlier study, RIG-I and MDA5, the two key factors up-stream of MAVS, were up-regulated in human dendritic cells phagocytosing CVB3-infected islets cells or treated with poly I:C. This indicats the underlying influence of IFNs or IFN inducers on MAVS related pathway [34]. Given the critical role of MAVS in IFN pathway, our results demonstrated the robust induction of both IFN-α and IFN-β in CVB3 infected HeLa cells over-expressing MAVS. Therefore, MAVS might be a potential target in therapy of the syndromes associated with CVB3 infection. Thus the factors either up-regulating MAVS expression or mediating the related immune response might be an alternative prescription in virus related syndromes.

## Conclusions

In summary, our results show that although CVB3 infection in HeLa cells induces type I IFNs, the anti-viral cytokines are insufficient to resist CVB3 replication. Introduction of exogenous MAVS protein into cells pre- or post- exposure to CVB3 helps to promote the activity of innate immune response and restrict the viral replication and viral caused cell damage. Thus, type I IFNs are involved in the antiviral responses.

## Methods

### Cells and viruses

HeLa cells were cultured in Dulbecco’s modified Eagle’s medium (DMEM; Gibco-BRL, Grand Island, N.Y., USA) supplemented with 10% fetal bovine serum (FBS; Biologica Industries, Kibbutz Beit Haemek, Israel) and 50 U/mL of penicillin and 50 mg/mL streptomycin. Cells were incubated at 37°C with 5% CO_2_ and separated for passage by 0.02% EDTA and 0.25% trypsin. CVB3-mCherry virus was kindly provided by Prof. Zhao-Hua ZHONG (Harbin Medical University). Briefly, the CVB3 variant expressing mCherry was constructed by insertion of the mCherry open-reading frame (ORF) at the 5’end of CVB3 ORF. This strategy for the construction of the recombinant CVB3 virus has been reported previously [35]. CVB3-mCherry and CVB3-woodruff strains were prepared in HeLa cells, maintained in DMEM (with 2% FBS) and harvested according to the established technique [35,36]. The virulence of CVB3-mCherry was 1.13 × 10^7^ plaque-forming unit (PFU)/mL and that of CVB3-woodruff is 1.0 × 10^8^ PFU/mL. CVB3 infection experiments were performed with MOI at the indicated dosage and time.

### Plasmids

The p-EGFP-N1 was purchased from Clonetech. The pcDNA3.1 vector and pcDNA3.1-FLAG-MAVS plasmid was a kind gift of Dr. Katherine Fitzgerald (University of Massachusetts Medical School) [37]. The MAVS ORF sequence was acquired by PCR amplification from FLAG-MAVS and cloned into the p-EGFP-N1 vector.

### Transfections

HeLa cells were seeded at 24 h before transfection or inoculation. Procedure of plasmid transfections were performed using Lipofectamine 2000 (Invitrogen) according to the Invitrogen’s protocol. Based on the guide, the adjusted plasmid dosage of transfection (80 ng/mL, 160 ng/mL, 320 ng/mL or 640 ng/mL) was used according to the requirement in experiments. Transfection was performed 24 h before exposure to CVB3 or just after viral absorption.

### Immunobloting

Transfected cells were harvested and resuspended with lysis buffer (Beyotime, China) followed by incubation at 4°C for 10 min and centrifugation at 12, 000 rpm for 15 min. The cell lysates were resuspended in sodium dodecyl sulfate (SDS) sample buffer (Sigma-Aldrich), separated by SDS-polyacrylamide gel electrophoresis (PAGE, 10% polyacrylamide gels), and transferred to a nitrocellulose membrane. The primary antibodies were purchased from Sigma-Aldrich (anti-FLAG) and Santa Cruz (anti-β-actin). The secondary antibody was the product of Zhongshan Golden Bridge-Bio, China. Specific signals were detected with an enhanced chemiluminescence system (Pierce) and ECL machine (LAS-3000, Fuji Japan).

### RT-qPCR

To evaluate the replication level of CVB3 and transcriptional level of type I Interferon and MAVS, cells with different treatment were harvested using RNAiso PLUS (TAKARA) at the indicated time and total RNA was extracted. Reverse transcription was performed using M-MLV (Invitrogen). Quantitative detection of CVB3, IFN-α/β and MAVS expression was performed with SYBGreen kit (TAKARA) reagent and Roche lightcycler 2.0 qPCR instrument (primers in Table 1). Fold variations between RNA samples were calculated by 2^-ΔΔct^ method after normalization to GAPDH [38].

**Table 1:** Sequences of primers

**Primers**	** Sequence**
IFN-α [39]	
F	5’- GGTGCTCAGCTGCAAGTCAA −3’
R	5’- GCTACCCAGGCTGTGGGTT −3’
IFN-β	
F	5’- AAGGCCAAGGAGTACAGTC −3’
R	5’- AGTTTCGGAGGTAACCTG −3’
GAPDH [40]	
F	5’- ATCACTGCCACCCAGAAGAC −3’
R	5’- TTTCTAGACGGCAGGTCAGG −3’
CVB3	
F	5’-GCACACACCCTCAAACCAGA-3’
R	5’-ATGAAACACGGACACCCAAAG-3’
MAVS	
F	5’-CTCCTCGCTGCGGGAAGGGT-3’
R	5’-GGGACGGTCCGAGGTCCCTG-3’

### Quantification of IFN-β by ELISA

Human IFN-β was assayed using the VeriKine Hmman IFN-β ELISA kit (PBL interferon source, USA), instruction.

### Fluorescence imaging

After HeLa cells were transfected with p-EGFP-MAVS and control plasmids and challenged with CVB3-mCherry, MAVS-GFP (in green) and mCherry (in red) in the HeLa cells were observed by fluorescent microscope.

### Cell staining

Cells in culture plate were treated as required in experiment and washed with PBS 3 times, each time for 5 min. Then cells were fixed to the plate by paraformaldehyde for 15 min at room temperature and washed as described. One drip of crystal violet was used to stain the nucleus of the cells followed by 3 washes in PBS. The cell nucleus stained purple.

### MTT test

HeLa cells were seeded at 5000 per well in 96 wells plate, cultured overnight, and transfected with plasmids and inoculated with MOI grades (MOI = 0, 10^-3^, 10^-2^, 10^-1^, 1) of CVB3. Forty-eight hours after treatment, sterile MTT reagent was added into culture supernatant at 20 μL per well. The supernatant was then discarded and DMSO at 100μL per well was added after incubation at 37°C with 5% CO_2_ for 6 h. The sample was vibrated for 10 min to dissolve the formed crystal. The absorbance was determined at 490 nm on microplate reader.

### Data analysis

For analysis of CVB3 replication and the expression of IFNs and MAVS, group data were expressed as mean ± s.e.m. The Student’s *t* test was used to evaluate differences between two groups. Statistical analyses were carried out using SPSS 13.0 statistical software. All statistical tests were two-sided. A *p*-value of < 0.05 and < 0.01 was taken as statistically significant.

## Abbreviations

MAVS: Mitochondrial antiviral signaling; CVB3: Coxsackievirus B3; IFN: Interferon; CAR: Coxsackievirus and adenovirus receptor; EGF: Enhanced green fluorescent protein; poly I:C: Polyinosine-polycytidylic acid; p.i: Post-infection; p.t: Post-transfection; ELISA: Enzyme linked immuno sorbent assay; PFU: Plaque forming units; IKK: IkappaB-related kinases; TBK1: TANK binding kinase 1; MOI: Multiplicity of infection; RIG-I: Retinoic-acid-inducible protein I; MDA5: Melanoma-differentiation-associated gene 5; IRF3: Interferon regulating factor 3; NFκB: Nuclear factor kappa B.

## Competing interests

The authors declare that they have no competing interests.

## Authors’ contributions

QMZ and WQS performed viral inoculations, RT-realtime PCR and ELISA. QMZ and YJL were responsible for western blot analysis. JMH participated in cell culture and preparation. WQS and YX expanded the CVB3 with viral plaque assay. JYZ and AXZ carried out the cell staining and imaging. JQ carried out the MTT test. AML performed data analysis. JW designed the primers for RT and qPCR. FMZ, LLW, QMZ and WQS conceived and designed the study and prepared the first draft of the article. All authors read and approved the final version of this manuscript.
